# Anthocyanin-Rich New Zealand Blackcurrant Extract Supports the Maintenance of Forearm Blood-Flow During Prolonged Sedentary Sitting

**DOI:** 10.3389/fnut.2020.00074

**Published:** 2020-05-27

**Authors:** Matthew J. Barnes, Blake G. Perry, Roger D. Hurst, Dominic Lomiwes

**Affiliations:** ^1^School of Sport, Exercise and Nutrition, Massey University, Palmerston North, New Zealand; ^2^School of Health Sciences, Massey University, Wellington, New Zealand; ^3^The New Zealand Institute for Plant and Food Research Ltd, Palmerston North, New Zealand

**Keywords:** anthocyanins, forearm blood flow, exercise, blackcurrant, hemodynamics

## Abstract

**Objectives:** We examined the acute effects of anthocyanin-rich New Zealand blackcurrant extract and a placebo on hemodynamics during 120 min of sedentary sitting in healthy males. Additionally, we investigated whether changes in resting hemodynamics altered repeated isometric hand-grip exercise performance and post exercise forearm blood flow (FBF).

**Methods:** Ten healthy males completed two trials during which they ingested either blackcurrant extract (1.87 mg total anthocyanins/kg bodyweight) or placebo powder. Heart rate, blood pressure and forearm blood flow were measured, and venous blood was sampled, prior to and 30, 60, 90 and 120 min-post ingestion. Participants remained seated for the duration of each trial. At 120 min post-ingestion participants completed as many repetitions of isometric hand-grip contractions as possible.

**Results:** Heart rate, blood pressure and mean arterial pressure changed over time (all *p* < 0.001) but did not differ between treatments. A treatment x time interaction for FBF (*p* = 0.025) and forearm vascular resistance (FVR) (*p* = 0.002) was found. No difference in the number of isometric hand-grip contractions was observed between treatments (*p* = 0.68) nor was there any treatment x time interaction in post-exercise FBF (*p* = 0.997). Plasma endothelin-1 (*p* = 0.023) and nitrate (*p* = 0.047) changed over time but did not differ between treatments (both *p* > 0.1). Plasma nitrite did not change over time (*p* = 0.732) or differ between treatments (*p* = 0.373).

**Conclusion:** This study demonstrated that acute ingestion of a single dose of blackcurrant extract maintained FBF and FVR during an extended period of sitting; however, this did not influence exercise performance during hand-grip exercise.

## Introduction

Sitting in an upright position for extended periods leads to significant changes in hemodynamics (1–3), as a result of blood pooling in the lower extremities and reduced venous return (4). Reduced limb blood flow, which accompanies prolonged periods of sedentary sitting, is associated with increased risk of thromboembolism and cardiovascular disease (1, 5). Therefore, nutrition-based interventions that maintain or improve blood flow during periods of prolonged inactivity/sitting may provide a viable and accessible means of minimizing the detrimental health effects of reduced blood flow and flow mediated dilatation.

Studies demonstrating the benefits of consuming berryfruit on cardiovascular health have ascribed these benefits to their high phytochemical and anthocyanin content (6, 7). These compounds are reported to influence vascular tone through their ability to regulate mechanisms that mediate nitric oxide bioavailability (8, 9) and endothelin-1 expression (10). Blackcurrants (*Ribes nigrum* L.) contain a range of different anthocyanins (11) and have been demonstrated to modulate resting forearm blood flow (12) and other hemodynamic variables (13) in healthy males. The size of these effects were dose dependent and coincided with the bioavailability of anthocyanins and their metabolites.

It is still unclear, however, whether blackcurrants can attenuate the detrimental changes in blood hemodynamics that occur during prolonged sitting. This is because nutritional intervention studies characterizing the effect of consuming berryfruit on resting measures of blood hemodynamics have not elaborated on whether participants remained seated in their resting position for the duration of the trial. This is a critical variable, as minor exercises (1, 3) or breaks (14) disrupt the hemodynamic changes that occur during prolonged sitting.

Given the evidence supporting the modulatory effect of blackcurrants on resting hemodynamics, there is interest in their potential as ergogenic aids for exercise performance. By altering arterial vessel tone, blackcurrant supplementation is proposed to enhance exercise performance through improved oxygen delivery, phosphocreatine resynthesis rates and lactate clearance (15). In a study by Cook et al. (16), New Zealand blackcurrant supplementation for seven days modulated blood flow at rest and during a single sustained submaximal isometric contraction, but did not affect muscular performance. In comparison, blackcurrant supplementation for the same duration was found to improve cycling trial time (17), running time to exhaustion (15), and sport climbing performance (18) in trained volunteers. Although hemodynamics were not measured in these latter studies, these findings highlight that increases in exercise performance gained from blackcurrant intake may be dependent on the mode of exercise.

While the potential ergogenic effects of blackcurrant extract have been investigated across a range of exercise modalities, it is currently unclear whether supplementation can benefit repeated isometric hand grip exercise, as occurs in a number of sports including rock climbing (19) and martial arts (20). Performance during repetitive isometric contractions of the forearm muscles is negatively impacted by muscle ischemia (21, 22) and the accumulation of lactate, and related acidosis (23). Intensity as low as 10 % of maximum voluntary isometric contraction (iMVC) force partially occludes forearm blood flow and can negatively impact muscle performance (21), while full occlusion occurs with intensities of 50–60% iMVC (24, 25); such forces are likely to occur during sports where the athlete grasps an opponent or supports their body weight during climbing, for example. The potential for handgrip performance to be improved by blackcurrant supplementation was shown by Potter et al. (18) who demonstrated an improvement in sport rock climbing performance after 7 days of supplementation, however it is unclear if any benefit is achieved with a single dose of blackcurrant extract and whether such improvement is related to changes in hemodynamics.

The objectives of this present study were to (1) investigate the hemodynamic effects, including changes in peripheral blood flow, of consuming a single dose of a blackcurrant extract during a prolonged period of sitting, and (2) identify whether a single dose of blackcurrant extract can alter repetitive, isometric handgrip performance. We hypothesized that the intake of blackcurrant extract would improve peripheral blood flow, compared with a placebo, and that this would improve muscular performance during fatiguing exercise.

## Materials and Methods

Ten healthy males (mean ± SD; age = 29.2 ± 6.0 years; mass ± 92.1 ± 19.9 kg; height = 181.6 ± 8.9 cm) volunteered to participate in this study. Participants were non-smokers and were free of cardiovascular disease and conditions that may affect circulation or blood flow. Each participant gave informed written consent and completed health screening prior to being familiarized to the experimental procedures. Ethical approval was obtained from the University's Human Ethics Committee.

### Nutritional Intervention

For this study, we used a commercial anthocyanin-rich extract made from New Zealand grown blackcurrants, sourced from the New Zealand Blackcurrant Co-operative (Nelson, New Zealand). The total anthocyanin composition of the blackcurrant powder was determined by high performance liquid chromatography (HPLC) using procedures described by Lyall et al. (26). Quantitative analysis indicate that the blackcurrant extract powder contained 37% (w/w) total anthocyanins with delphinidin-3-rutinoside (44%), delphinidin-3-glucoside (13%), cyanidin-3-rutinoside (39%), and cyanidin-3-glucoside (4%) making up the total anthocyanin composition present in the extract.

The blackcurrant extract was encapsulated in opaque gelatin capsules and were served to trial participants at a dose of 1.87 mg total anthocyanins/kg bodyweight (172.23 ± 37.21 mg total anthocyanins). This dose was based off the findings of Matsumoto et al. (12), who used 1.84 mg anthocyanins /kg bodyweight, and pilot work using this specific blackcurrant extract. The sugar placebo powder intervention was prepared to contain the equivalent amounts of glucose (20%), fructose (24.2%), and sucrose (3.3%) present in the dose of blackcurrant extract and encapsulated in gelatin. Both blackcurrant extract and placebo interventions were consumed directly from an opaque container with a maximum of 250 mL water.

### Experimental Design

Participants underwent a familiarization and two subsequent trials, each separated by at least 1 week, in which the hemodynamic response and performance effects of blackcurrant extract were compared to the responses with the placebo at rest and during exercise. Allocation of treatment was double-blinded and randomly assigned so that an equal number of participants were allocated in each treatment intervention in the first trial. Participants were subsequently allocated to the opposite treatment in the second trial. Prior to each trial, participants were instructed to abstain from caffeine, alcohol, exercise and foods containing anthocyanins in the 24 h period; participants were provided with a list of foods to avoid.

At least 1 week after familiarization, having fasted overnight and having consumed a standardized pre-trial meal (8 g protein, 45 g carbohydrates, 11.4 g fat; 1,450 kJ) and bolus of water (600 mL) 120 min prior, participants presented to the laboratory in the morning. To ensure consistency, the time of day was the same between trials for each participant. Participants were seated in an upright position with knee and hip angles at ~90° and both arms supported, at approximately heart height, on a cushioned table. Participants sat at rest for 30 min before a blood sample was collected into an EDTA vacutainer from the antecubital vein of the upper arm and baseline measures of forearm blood flow (FBF), forearm skin temperature, heart rate (HR), systolic (SBP), and diastolic (DBP) blood pressure were made. Participants then consumed their allocated treatment intervention, containing either blackcurrant extract or placebo. Participants then remained seated for 120 min and the same measures were made every 30 min, except blood sampling which occurred again at 120 min only.

Having sat for 120 min, participants completed a bout of intermittent hand-grip exercise to volitional fatigue. The number of repetitions completed was recorded. Forearm blood flow of the exercised arm was subsequently measured immediately after (0), 2, 5, 10, and 15 min post-exercise.

### Hemodynamic Measures

Blood pressure was measured non-invasively via finger photoplethysmography (FinaPres® Medical Systems: Biomedical Instruments, Amsterdam) and periodically checked against a manual sphygmomanometer for accuracy. HR was measured with 3-lead electrocardiogram (ADInstruments Ltd., Australia) and cardiac output $\dot{Q}$ was calculated using Beatscope 1.02 software (Biomedical Instruments) from the blood pressure waveform using the Medflow technique (27).

Forearm blood flow was measured using standard procedures for venous occlusion plethysmography (28). A mercury-in-silastic double strand gauge (Hokanson®, Bellavue, USA) was placed around the widest region of the forearm of the dominant hand and changes in forearm circumference were recorded in LabChart for Windows (v7, ADInstruments Ltd.) via a custom made amplifier and PowerLab® data acquisition system (ADInstruments Ltd.). A 6 x 83 cm tourniquet cuff (Hokanson®) was placed around the upper arm, just proximal to the elbow, and rapidly inflated to 40 mmHg by a custom-made air compressor. Pressure was applied for 10 s before being slowly released.

Forearm arterial blood flow (mL/100 mL/min) was then calculated using the following equation:

200 x increase in forearm circumference (mm/min) / resting forearm circumference (mm)

$$\begin{array}{l} \frac{200\;\times \text{ increase in forearm circumference }\left(\text{mm}/\text{min}\right)}{\text{resting forearm circumference}} \end{array}$$

Total peripheral resistance (TPR) and forearm vascular resistance (FVR) were calculated using the following equations:

$$\begin{array}{l} \text{TPR}=\;\frac{\text{Mean arterial pressure}}{\left(\dot{\text{Q}}\right)\;}\\ \text{FVR}=\;\frac{\text{Mean arterial pressure}}{\text{Forearm blood flow}}\; \end{array}$$

To reduce the influence of changes in forearm skin temperature on FBF, attempts were made to maintain forearm skin temperature at 30°C. Consistent forearm skin temperature was maintained using a liquid conditioning garment (CoreTech® tube suit, Delta Temax Inc., Canada) worn on the arm of interest; this garment was fitted and forearm skin temperature adjusted and maintained at the target temperature from 30 min prior to baseline measures until the completion of the trial. Temperature (accurate to 0.1°C) and flow rate (15.7 L/min) of the circulating liquid was controlled and pumped using a water heater/cooler with a built-in pump (Neslab Instruments Inc., USA). Skin temperature was recorded with a skin temperature probe (ADInstruments Ltd.) taped on the forearm, 2 cm below the position of the mercury strain gauge. Ambient temperature and relative humidity were maintained at 21 ± 0.3°C and 55 ± 2%, respectively, for all trials.

### Intermittent Hand Grip Exercise

Having sat at rest for 150 min, participants completed as many repetitions as possible at 60% of their iMVC force, obtained during familiarization, by squeezing a grip force transducer (MLT004/ST, ADInstruments Ltd., Australia) with their dominant hand. Each repetition was held for 5 s at the target force, with 10 s rest between efforts; this was continued until participants could no longer match the specified force output over 3 consecutive repetitions. Grip force output was collected using a PowerLab data acquisition system and recorded and displayed in LabChart (ADInstruments Ltd.) so that participants had real-time visual feedback of their force output. The number of repetitions completed, excluding the 3 consecutive repetitions below the target force was compared between treatment interventions.

### Plasma Nitrite and Nitrate

Quantification of endogenous nitrite and nitrate levels in plasma samples, taken prior to and 120 min after treatment ingestion, was performed using a commercially available kit (SKGE001, RnD Systems™), according to the manufacturer's instructions. Endogenous nitrite was quantified by measuring sample and standard absorbance at 540 nm (Fluostar® Omega plate reader, BMG Labtech, Ortenberg, Germany) following the addition of Griess reagents. For nitrate quantification, plasma nitrate was enzymatically converted to nitrite with nitrate reductase followed by quantification of total nitrite as per the manufacturer's instructions. Nitrate concentrations were then calculated by subtracting the endogenous nitrite concentration from total nitrite concentration. Samples were analyzed in duplicate with a coefficient of variation of 5.62%.

### Endothelin-1

Plasma endothelin-1 concentration was measured in samples collected before and 120 min after treatment ingestion using a commercially available endothelin-1 (human) enzyme immunoassay kit (ADI-900-020A, Enzo® Life Sciences). Plasma samples initially were diluted 1:4 in the kit's assay buffer endothelin-1 quantification. Samples were analyzed in duplicate with a coefficient of variation of 11.1%.

### Statistical Analysis

Statistical analysis was performed in IBM® SPSS Statistics v21 (NY, USA). Two-way (treatment x time) repeated measures analysis of variance (ANOVA) was carried out to investigate changes in blood measures, hemodynamic variables and handgrip performance. Where significant main effects were identified, *post hoc* analysis using Bonferroni adjustment was made. Analysis was performed on raw data and, where indicated, as a percentage change from baseline. Residual plots were inspected to ensure that the normality and homogeneity of variance assumptions for ANOVA were met and as FBF did not meet these assumptions, values were log-transformed to stabilize the variance.

To compare the reliability of baseline measures of FBF, the intraclass correlation coefficient (ICC) and 95% confidence intervals (CI) were calculated with a two-factor mixed-effect model with absolute agreement. Reliability was classified as poor (ICC <0.5), moderate (0.5 to 0.75), good (0.75 to 0.9) and excellent (> 0.9) (29). Significance level was set at *p* ≤ 0.05.

## Results

### Central Hemodynamic Changes

**Figure 1:**
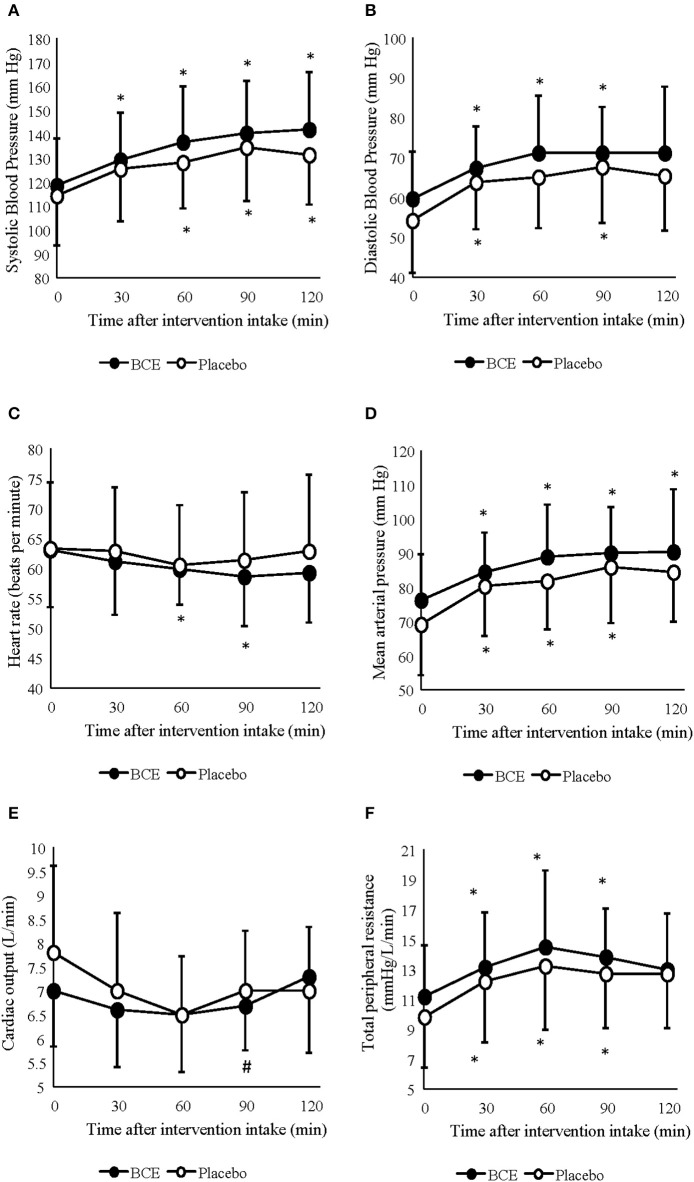
Acute effects of consuming either blackcurrant extract (BCE) and placebo powder on resting **(A)** systolic blood pressure, **(B)** diastolic blood pressure, **(C)** heart rate, **(D)** mean arterial pressure, **(E)** cardiac output and **(F)** total peripheral resistance. Results are expressed as mean ± SD. *indicates significant difference from baseline evaluation (*p* < 0.05); ^#^indicates significant difference from 60 min within interventions (*p* < 0.05).

A significant increase in SBP (*p* < 0.001) and DBP (*p* < 0.001) pressure was observed during the 120 min period at rest (Figures 1A,B). Changes in SBP occurred sooner in the blackcurrant extract intervention during rest, with a significant increase in systolic blood pressure, relative to baseline values, at 30 min after blackcurrant extract intake. However, no difference between treatment groups was found (*p* = 0.113 and 0.119, respectively).

Mean arterial pressure (MAP) and TPR also significantly increased over the 120 min period at rest (*p* = 0.001 and 0.0001, respectively) (Figures 1C,D). No difference between treatment interventions was found for either parameter (*p* = 0.085 and 0.160, respectively). $\dot{Q}$ changed over time (*p* = 0.003) but did not differ between treatments (*p* = 0.367) (Figure 1E). A change in HR was also observed over time (*p* < 0.001); however, no difference was seen between treatments (*p* = 0.178) (Figure 1F). No treatment x time interactions were observed for any of the central hemodynamic variables measured in this study.

### Forearm Blood Flow and Skin Temperature Changes

**Figure 2:**
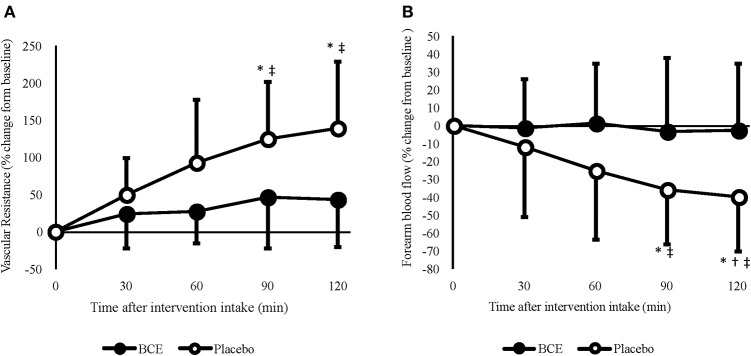
Acute effects of consuming blackcurrant extract (BCE) and placebo powder on forearm **(A)** vascular resistance and **(B)** blood flow. Values are percentage change from baseline levels (0 min) and expressed as mean ± SD. *indicates significant difference from baseline values, significant difference from 30 min (*p* = 0.039) and ^‡^indicates significant difference from BCE (*p* < 0.05).

Irrespective of the experimental controls put in place prior to each trial, significant differences in FBF between treatments were found at baseline (blackcurrant extract 2.49 ± 1.27 vs placebo 2.84 ± 4.29 mL/100 mL/min; *p* < 0.001). Therefore, FBF was analyzed as both raw (log-transformed) and normalized (percentage change from baseline) values (Figure 2B). Moderate reliability was found between baseline measures of FBF [ICC (95% CI) = 0.761 (-0.154–0.946)]. No effect for order (trial 1 vs trial 2) in this crossover study was found for any of the FBF measures (all *p* > 0.5).

A treatment x time interaction was found for both raw (*p* = 0.036) and percentage change FBF (*p* = 0.005). No change in FBF was observed with blackcurrant extract; however, after 90 and 120 min, FBF was significantly reduced with the placebo (90 min = −35.8 ± 9.6%, *p* = 0.029; 120 min = −39.4 ± 9.7, *p* = 0.018). Although a reduction in FBF was only evident with the placebo, the difference in baseline values between the treatment groups meant that FBF was not significantly different between treatments at 60 min (blackcurrant extract 2.4 ± 0.4 vs. placebo 3.0 ± 0.5 mL/100 mL/min; *p* = 0.329), 90 min (blackcurrant extract 2.2 ± 0.3 vs placebo 2.6 ± 0.4 mL/100 mL/min; *p* = 0.504) and prior to hand-grip exercise at 120 min (blackcurrant extract 2.2 ± 0.2 vs. placebo 2.4 ± 0.4 mL/100 mL/min; *p* = 0.932).

A time effect (*p* = 0.040) was found for FVR (Figure 2A); however, no other significant effects were seen. When individual's data were normalized, as percent change from baseline, FVR increased significantly over time (*p* = 0.001) and differed between treatments (*p* < 0.001). A significant treatment x time interaction was also observed (*p* = 0.002). *Post hoc* analysis revealed that, similar to FBF, FVR did not change over time with blackcurrant extract. However, FVR significantly increased after 90 min (124.8 ± 24.1%, *p* = 0.006) and 120 min (138.9 ± 28.1%, *p* = 0.008) with the placebo.

Mean forearm skin temperature was 30.3 ± 1.6°C. No change in forearm skin temperature was observed over the 120 min rest period (*p* = 0.170), and no difference in temperature was evident between treatments (*p* = 0.713). Furthermore, no treatment x time interaction was detected (*p* = 0.790).

### Blood Parameters

**Figure 3:**
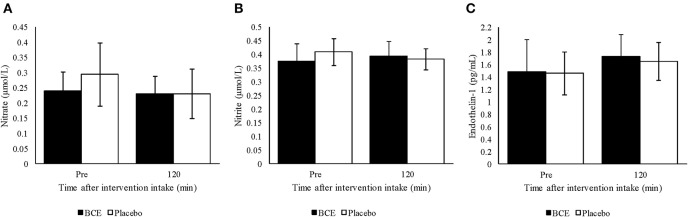
Plasma **(A)** nitrite, **(B)** nitrate and **(C)** endothelin-1 pre and 120 min after ingesting a single dose of either blackcurrant extract (BCE) or placebo powder. Data are mean ± SD.

No treatment or time effect (*p* = 0.373 and 0.732, respectively) was observed for plasma nitrite from samples measured prior to and 120 min after either blackcurrant extract or placebo ingestion (Figure 3A). Additionally, no treatment x time interaction was found (*p* = 0.144). A general decline in plasma nitrate concentration was detected over time (*p* = 0.047). Although a greater decrease in plasma nitrate concentration was observed with the placebo than with the blackcurrant extract, no treatment effect or treatment x time interaction was observed (*p* = 0.141 and 0.127, respectively) (Figure 3B).

Endothelin-1 concentration in plasma tended to be higher 120 min after intervention intake compared to baseline concentration (*p* = 0.023) (Figure 3C). However, no treatment effect (*p* = 0.561) or treatment x time interaction was found (*p* = 0.812).

### Exercise Performance

No difference in the number of repetitions completed was evident between trials (blackcurrant = 74 ± 29 repetitions vs placebo = 77 ± 45 repetitions, *p* = 0.68). A similar decrease in force (blackcurrant extract = −16.5% vs. placebo = −16.56 %, *p* = 0.98) was observed when comparing the average force over the first five repetitions (blackcurrant extract = 31.7 ± 5.3 kg, placebo = 31.9 ± 5.7 kg) with the last five repetitions (blackcurrant extract = 26.5 ± 4.8 kg, placebo = 26.6 ± 4.9 kg) between treatments.

### Post Exercise Forearm Blood Flow

**Figure 4:**
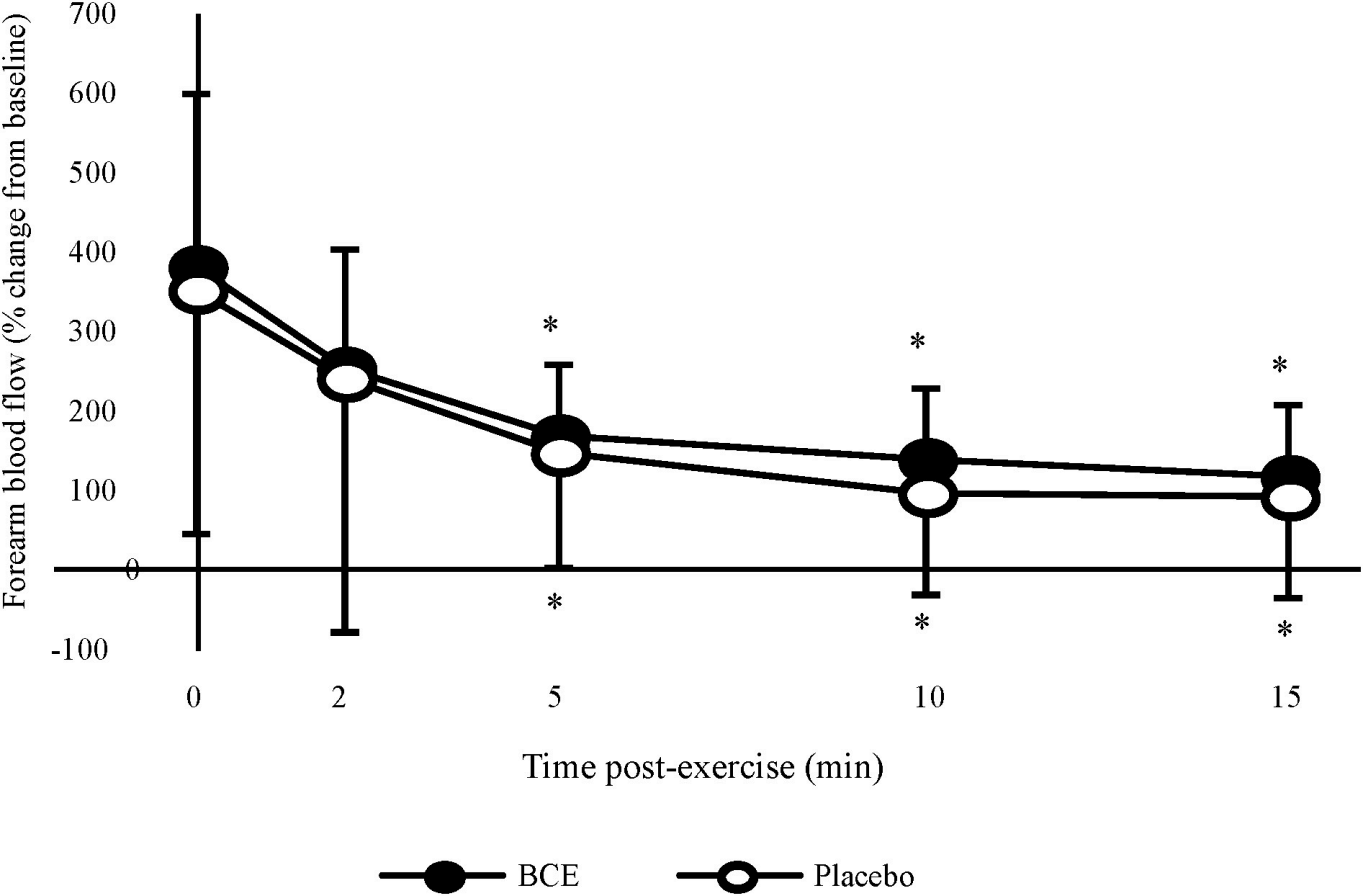
Percentage change, from baseline, in forearm blood flow (FBF) after a bout of intermittent hand-grip exercise 2-h after ingesting blackcurrant extract (BCE) or placebo powder. Data are mean ± SD. * indicates significant difference from FBF measures immediately post-exercise (0 min) (*p* < 0.05).

Absolute post-exercise FBF results showed a significant treatment (*p* < 0.001) and time (*p* = 0.001) effect with FBF declining post-exercise and tending to be higher with PP. However, no treatment x time interaction was observed. When participants' post-exercise FBF were normalized to their baseline values (Figure 4), a significant time effect (*p* < 0.001) was observed with FBF progressively declining over the 15 min post-exercise recovery period. No treatment effect (*p* = 0.347) or treatment x time interaction was detected (*p* = 0.997).

## Discussion

The primary aim of this study was to investigate the effects of blackcurrant extract on FBF, and other hemodynamic measures, at rest during a prolonged period of sitting and after exercise. Additionally, intermittent handgrip exercise was performed to determine whether exercise performance increased with blackcurrant extract, compared with a sugar-equivalent placebo control. Our findings show that a single dose of blackcurrant extract maintained both FBF and FVR during prolonged sitting but did not enhance resistance to fatigue from repeated submaximal isometric hand contractions.

Uninterrupted sitting for 120 min also led to significant increases in blood pressure (SBP and DBP), MAP, $\dot{Q}$ and TPR in both intervention groups. These trends are comparable with previous studies that have reported increases in DBP, MAP, and TPR in healthy men following prolonged sitting exceeding one hour (4, 30). These changes are indicative of blood pooling and reduced blood flow in the lower extremities that is characteristic of prolonged sitting (4). It has been proposed that venous distention (31) and sympathetic nerve activity (32) during prolonged sitting induce arterial vasoconstriction, attenuating FBF.

Epidemiological and clinical studies demonstrating the benefits of consuming berryfruit on cardiovascular health have ascribed these benefits to their high anthocyanin content (6, 33). Blackcurrants are rich in anthocyanins, with > 97% of total anthocyanins in blackcurrants comprising of delphinidin and cyanidin glycosides (11). Dietary anthocyanins are rapidly absorbed, with concentrations of their glycosylated forms peaking in blood plasma 1 h after ingestion, then declining thereafter (34). The bioavailability of anthocyanin compounds and their metabolites have been reported to parallel modulation in resting blood flow and cardiovascular function (12, 33).

Although great care was taken to collect data in a highly controlled manner, including standardizing diet, time of day, ambient and skin temperature, arm position and restricting previous day physical activity, a significant difference in FBF was found between treatments at baseline. However, the controls put in place in the present study appear to have been successful as no order effect was found between trial 1 and trial 2. Irrespective of controls put in place, previous studies investigating FBF, using venous occlusion plethysmography, have reported considerable variability between days (35–38). For example, Alomari et al. (35) reported ICC of 0.29 when FBF was measured on different days, this value is much lower than the reliability (ICC = 0.761) of baseline FBF in this study. This common variability lead Alomari et (35) to suggest that a “wandering” baseline exists which may make it difficult to directly compare measures at rest.

In the present study, 120 min of uninterrupted sitting led to the progressive decline in FBF. Importantly, we show that the decline in FBF following 90 and 120 min of uninterrupted sitting was mitigated by consuming a single dose of blackcurrant extract. The progressive increase in FVR during the 120 min sitting period was similarly moderated by blackcurrant extract so that FVR did not significantly rise above baseline levels. Taken together, these findings support the efficacy of blackcurrant extract for maintaining forearm blood hemodynamics during prolonged sitting. Although anthocyanin bioavailability was not measured in this study, the time points where treatment differences in FBF and FVR were observed corresponded with the expected bioavailability of blackcurrant anthocyanins (39).

These findings align with similar placebo-controlled research (12) reporting significantly higher resting FBF 120 min after consuming a similar amount of blackcurrant derived anthocyanins (1.84 mg/kg), compared with a placebo intervention. It should be noted, however, that Matsumoto et al. (12) did not explicitly disclose whether volunteers remained seated in their resting position for the duration of the trial period. This is important to emphasize as minor breaks and exercise both reverse hemodynamic changes induced from prolonged sitting (1, 3).

Existing studies suggest that the efficacy of flavonoids in modulating FBF during prolonged sitting is independent of secondary hemodynamic parameters. Consuming a single dose of anthocyanin-rich blackcurrant extract and blueberry juice has been shown to have no observable effects on resting blood pressure and HR, despite reported increases in FBF and endothelial function, respectively (12, 33). In the present study, the increase in SBP and MAP from baseline levels after 30 min of sitting remained elevated for the duration of the 120 min with the blackcurrant extract treatment. This highlights the potential for blackcurrant extract to assist the early onset and maintenance of these hemodynamic compensatory mechanisms during sitting, thereby maintaining FBF and FVR.

Declining concentrations of nitric oxide, a key vasodilator that regulates cardiovascular function, contributes to venous stasis during prolonged sitting (3). This may be due to the reduction in the phosphorylation of eNOS, which catalyzes the production of NO from L-arginine and NADPH. There is growing evidence for the bio-efficacy of berry-derived polyphenols increasing NO bioavailability, thus regulating blood hemodynamics. Blackcurrant concentrate, containing up to 16.25 μg of anthocyanin, has been demonstrated to change vasorelaxation *ex vivo*, in a dose dependent manner, through mechanisms that upregulate nitric oxide synthesis (9). Similarly, blueberry consumption is purported to increase NO bioavailability through the inhibition of neutrophil NADPH oxidase activity, thus sparing NO from being converted to superoxide (33). As NO was not measured in these previous studies it is difficult to make direct comparison to our findings, however evidence for the role of berry-derived anthocyanins in increasing NO bioavailability is compelling. Our results indicate that blackcurrant extract intake did not alter nitric oxide bioavailability, as indicated by negligible changes in plasma nitrite and nitrate levels. A possible explanation for the apparent disparity between our findings and those reported by Nakamura et al. (9) may be due to the significantly lower anthocyanin dose used in this study. Nakamura et al. (9) directly treated rat aorta tissue with a concentration of between 10 and 25 μg/mL of anthocyanin, however, the concentration of bioavailable anthocyanins in the present study is likely to be at the nM (39) not the mM concentration used by Nakamura et al. (9).

Prolonged sitting has been proposed to induce oxidative stress which favors the up-regulation of endothelium-derived endothelin-1 (3). Although the obvious effect of this potent vasoconstrictor on vascular tone is apparent, animal studies indicate endothelin-1 upregulation may also lead to reduced nitric oxide bioavailability via NADPH oxidase activation and inhibition of eNOS activity (40, 41). There is increasing evidence supporting the efficacy of flavonoid compounds to maintain vascular function through their ability to down-regulate endothelin-1. Anthocyanidins have been demonstrated to reduce endothelial-derived endothelin-1 expression while up-regulating eNOS protein expression *in vitro* (10). Similarly, quercetin and (-)-epicatechin acutely reduced plasma endothelin-1 and increased circulating nitric oxide in healthy individuals (42). Prolonged sitting for the duration used in this study did not lead to any modulation in plasma endothelin-1. Taken together, our findings indicate that the efficacy of blackcurrant extract in mitigating forearm hemodynamic changes during 120 min of uninterrupted sitting is independent of endothelin-1 expression and circulating nitric oxide concentration.

The potential for New Zealand blackcurrants to improve isometric muscular performance was demonstrated by Cook et al. (16), who reported an increase in femoral artery diameter during a single sustained isometric contraction (30% iMVC) following seven days of blackcurrant supplementation. Unlike the low intensity contraction model applied by Cook et al. (16), which did not appear to limit blood flow to the quadriceps, the intensity used in this study (60% iMVC) is likely to occlude FBF during each contraction (24, 25); therefore, blood flow is likely to be important for sustaining muscular performance and delaying the onset of fatigue. Despite differences in relative FBF and FVR between treatment groups at the commencement of the handgrip exercise, no difference in exercise performance was observed between treatment groups. The absence of discernible improvement in exercise performance, from blackcurrant extract intake, may be due to the lack of difference in absolute FBF between treatments at the start of the hand-grip exercise, as a result of differences in baseline FBF and treatment related changes occurring over 120-min of sitting. Additionally, the metabolic stimulus provided by repetitive muscular contractions may have resulted in maximal vasodilation and therefore any further vasodilation, due to blackcurrant extract, may not have been possible. Given these limitations, it is not currently possible to deduce whether acute ingestion of a single dose of blackcurrant extract alters performance during repetitive, submaximal hand-grip exercise.

Hyperemia to exercising muscles rapidly declines after the cessation of exercise. The rate of this decline is dependent upon exercise intensity so that greater elevations in FBF occur following heavier workloads, even after considerable recovery time (43, 44). In addition, elevated blood flow at higher workloads corresponds with higher oxygen uptake in the recovering muscle. This increased oxygen demand may be an adaptive response to assist muscle recovery through the conversion of lactate to glycogen and resynthesis of ATP and creatine phosphate utilized during exercise (45, 46). Nutritional supplements that promote vasodilation, through increasing nitric oxide bioavailability, are proposed to improve oxygen and nutrient delivery to exercised muscles and augment hypertrophy in exercise muscle tissues leading to increased exercise performance and recovery (47). With growing evidence demonstrating the efficacy of berry anthocyanins to modulate blood hemodynamics, it is plausible for blackcurrant anthocyanins to support exercise recovery through their potential to promote post-exercise blood flow. In this study, however, blackcurrant extract supplementation did not lead to an observable increase in FBF during 15 min of exercise recovery despite mitigating the decline in FBF at rest.

Although great care was taken in the control of this study, there are several limitations, in addition to those previously discussed, that should be considered. Firstly, this study was only done on healthy males with no apparent cardiovascular disease. Ovarian sex hormones produce known changes in vascular tone with estrogen promoting vasodilation via nitric oxide dependent and independent pathways (48). Furthermore, participants in the current study had no previous history of vascular disease and were non-smokers. Vascular diseases [e.g., atherosclerosis (49)] and life-style factors, such as smoking (50) modify resting vascular tone and regulation via nitric oxide. The results of the current study may not be applicable to eumenorrheic females during high estrogen phases of the menstrual cycle (late follicular) and to clinical cohorts where modified vascular regulation is apparent.

Secondly, while the measure of forearm blood flow using venous occlusion plethysmography is considered the “gold standard” for assessing vascular function, particularly in the forearm vascular bed (51), additional measures of arterial stiffness and muscle perfusion may have provided further insight into the effects of blackcurrant extract on vascular function.

In summary, ingestion of a single dose of a New Zealand blackcurrant extract preserved forearm blood flow during a prolonged period of sitting and inactivity. This finding supports the growing evidence that blackcurrant anthocyanins have the ability to alter hemodynamics during rest/inactivity. This may provide health benefits to those unable to move their lower limbs regularly, such as individuals undertaking long-haul air travel, sedentary work, or those with decreased mobility due to illness and disease. However, further research is needed to better understand the implications of altered hemodynamics on lower limb blood flow, as the effects of black currant extract/anthocyanins are likely to be systemic in nature, and not just localized to the upper limbs. Although blood flow was maintained, this did not improve intermittent hand-grip exercise; however, because of experimental limitations, further research is needed to identify whether blackcurrant anthocyanins can improve repetitive muscular work.

## Data Availability Statement

The datasets generated for this study are available on request to the corresponding author.

## Ethics Statement

The studies involving human participants were reviewed and approved by Massey University Human Ethics Committee, Southern A. The patients/participants provided their written informed consent to participate in this study.

## Author Contributions

All authors conceptualized and designed the study. MB, DL, and BP recruited the participants, performed the human trials, carried out the experimental analysis on collected blood samples, the interpretation of results and drafted the manuscript. RH reviewed the manuscript and approved the final version submitted.

## Conflict of Interest

DL and RH were employed by company The New Zealand Institute for Plant and Food Research Ltd. The remaining authors declare that the research was conducted in the absence of any commercial or financial relationships that could be construed as a potential conflict of interest.
